# Information rather than architecture: cross-learner attribution and clinician–model complementarity in CIN2+ risk prediction for women referred for colposcopy

**DOI:** 10.3389/fonc.2026.1814215

**Published:** 2026-10-30

**Authors:** Yuzhang Wu, Aihong Wang

**Affiliations:** 1 Department of Telecommunications Engineering and Management, Beijing University of Posts and Telecommunications, Beijing, China; 2 School of Electronic Engineering and Computer Science, Queen Mary University of London, London, United Kingdom; 3 Department of Obstetrics and Gynaecology, The Second Affiliated Hospital of Shandong First Medical University, Tai’an, Shandong, China

**Keywords:** cervical intraepithelial neoplasia, clinical prediction model, clinician–model complementarity, colposcopy, decision curve analysis, logistic regression, Shapley value, tabular deep learning

## Abstract

**Background:**

Deep tabular models, including self-supervised and pretrained foundation models, are increasingly proposed for clinical risk prediction, but their value over conventional learners is rarely tested against matched controls, and their role relative to clinician judgement is seldom examined.

**Methods:**

In a retrospective single-centre cohort of 879 women referred for colposcopy (242 CIN2+, 27.5%), 15 routinely recorded variables, including a clinician image-based assessment documented before histopathology, were modelled with ten model families under one fixed protocol (single fold assignment and seed, no tuning). Discrimination was compared by paired DeLong tests and bootstrap. Two analyses were pre-specified: exact Shapley attribution of discrimination to five clinical groups of variables in every model family, and a complementarity analysis asking whether routine data identify CIN2+ among women judged negative by the clinician. Frozen models were evaluated in a 103-case two-centre external cohort.

**Results:**

Out-of-fold AUROC ranged from 0.726 to 0.761. No model outperformed logistic regression (AUROC 0.757; CatBoost −0.002, p = 0.860; TabPFN +0.004, p = 0.554), and three matched ablations of a self-supervised tabular Transformer were null (all p ≥ 0.096). Removing the clinician assessment lowered AUROC in all ten families (by 0.041 to 0.072; all p ≤ 0.008). Shapley attribution ranked five variable groups identically in every family (Kendall’s W = 1.00; permutation p < 0.001): the clinician assessment (39% to 50% of discrimination above chance), then cytology, HPV testing, colposcopic findings and demographic history. The clinician assessment alone had sensitivity 0.504 and specificity 0.882; 49.6% of CIN2+ occurred in clinician-negative women. Re-reviewing the 20% of clinician-negative women with the highest model risk identified 34.2% (95% CI 25.7–42.7%) of the missed CIN2+, against 20% expected at random, raising pathway sensitivity to 0.674 at specificity 0.733. External AUROC of logistic regression was 0.710.

**Conclusions:**

On routine structured data from a colposcopy-referral population, and under a common protocol, model architecture did not determine performance; predictor information did. Retrospectively, routine data complemented the clinician modestly in clinician-negative women, rather than replacing clinical judgement. Claims that complex architectures improve clinical tabular prediction should be tested against logistic regression with paired uncertainty across repeated data splits.

## 1 Introduction

Cervical cancer remains a leading cause of cancer death in women worldwide (1). Cervical intraepithelial neoplasia grade 2 or worse (CIN2+) is the conventional treatment threshold in cervical screening, and most management decisions after an abnormal screening result turn on the estimated risk of CIN2+ (2). Colposcopy is the pivotal step in that pathway, yet colposcopic impression is subjective and its sensitivity for CIN2+ is imperfect (3). Structured risk models built from demographic, human papillomavirus (HPV), cytology and colposcopic variables have therefore been proposed to support triage (4). The strongest reported models draw on large screening cohorts and full HPV genotyping (5); many clinical settings have neither.

Machine-learning methods are increasingly applied to such data. A systematic review found no performance benefit of machine learning over logistic regression for clinical prediction models (6). Benchmark studies show that tree ensembles remain difficult to beat on typical tabular data (7). Newer approaches, including tabular Transformers (8), self-supervised pretraining by feature masking (9) and tabular foundation models pretrained on synthetic priors (10), are reported to narrow or reverse that gap. On moderate-sized clinical tables, however, such claims often rest on comparisons between separately implemented pipelines and on single training runs, where variation between runs can be as large as the effects being claimed (11).

Two further questions are rarely addressed. The first concerns attribution: explanations such as integrated gradients or SHapley Additive exPlanations (SHAP) describe one fitted model, and their conclusions may change with the learner (12). Importance can instead be defined at the population level by refitting on subsets of variables (13), and whether it is a property of the data or of the model can then be asked directly by examining many learners at once. The second concerns clinical role. Datasets from colposcopy clinics often contain a near-decision variable recording the clinician’s own judgement. A model that includes it partly reproduces the clinician. The useful clinical question is then whether routine data add to the clinician, not whether a model can replace the clinician (14).

We report a controlled comparison with three aims: first, to compare ten model families, including a self-supervised tabular Transformer with masked feature modelling (Tab-MFM) and its matched ablations, against logistic regression with paired uncertainty; second, to quantify which clinical groups of variables drive performance and whether that answer is the same across learners; and third, to evaluate whether routine data complement the clinician’s assessment in a sequential referral pathway.

## 2 Materials and methods

### 2.1 Study design, setting and participants

This retrospective study used structured clinical records from the Second Affiliated Hospital of Shandong First Medical University. The development cohort comprised women evaluated between December 2024 and December 2025 who underwent colposcopy and had histopathology available as the reference standard. Of 887 screened records, 8 were excluded for a missing or invalid histopathology label, leaving 879 women. An independent external cohort of 103 women from two independent institutions, with no patient overlap, was analysed with the same endpoint and predictor definitions. The development and external cohorts have been described previously (15). The study design and analysis workflow are summarised in **Figure 1**. Reporting follows the TRIPOD+AI statement (16), and the risk-of-bias considerations of the Prediction model Risk Of Bias ASsessment Tool (PROBAST) and PROBAST+AI informed the design (17, 18).

**Figure 1:**
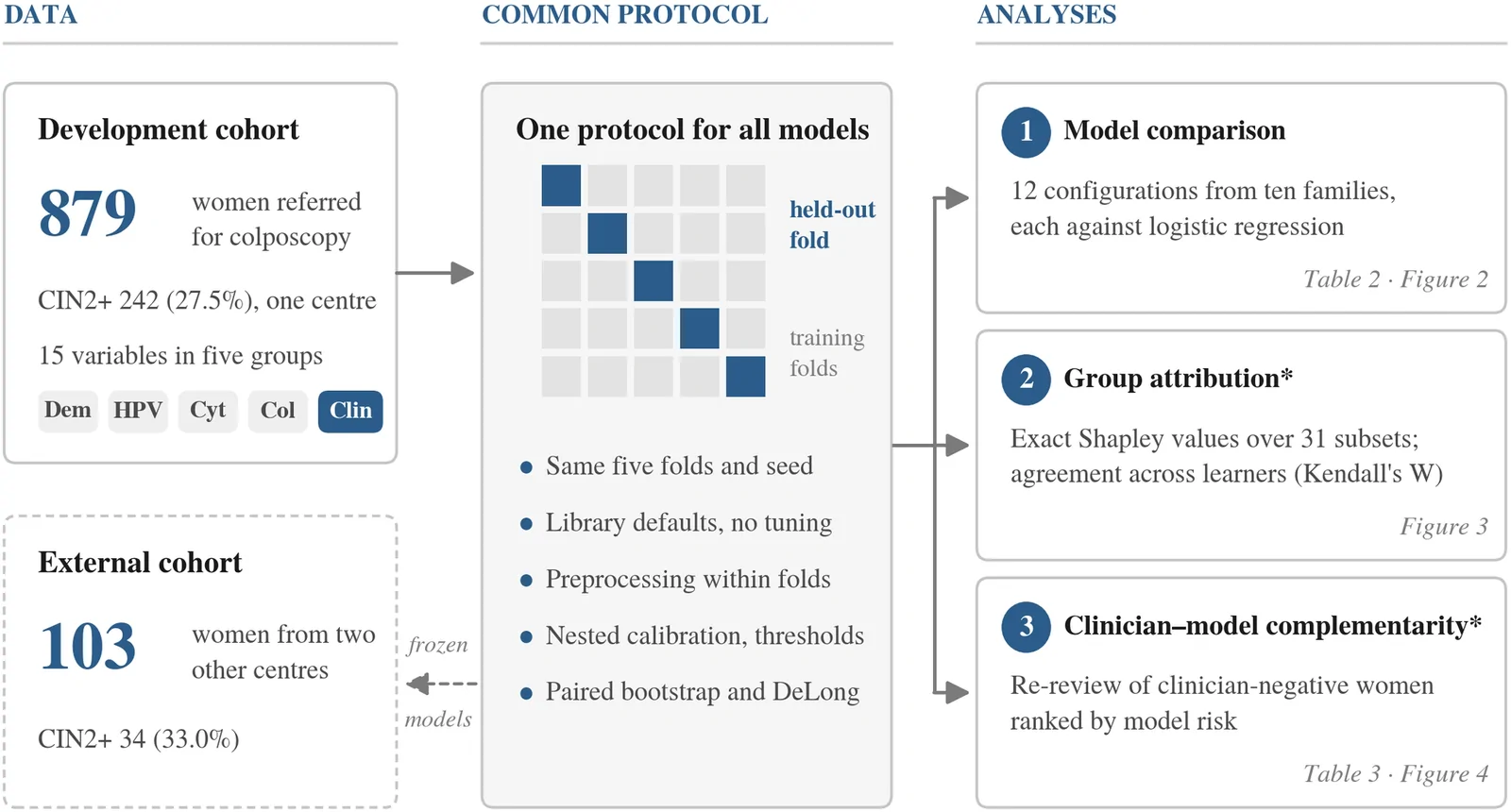
Study design and analysis workflow. Both cohorts, the protocol shared by every model and analysis, and the three analyses, with the tables and figures that report them. In the five-fold grid, dark cells mark the held-out fold in each split. The external cohort was evaluated descriptively with frozen models (Section 3.9); calibration, decision curves and learning curves are shown in **Figure 5**. *Specified in writing before computation. Dem, demographic and reproductive history; HPV, HPV testing; Cyt, cytology; Col, colposcopic findings; Clin, clinician image-based assessment.

### 2.2 Reference standard

The outcome was histopathologically confirmed CIN2+ after colposcopy. Colposcopy followed routine practice with acetic acid and iodine application; targeted biopsies were taken, with endocervical curettage when indicated, and when several specimens were available the most severe diagnosis defined the outcome. Histopathology was reviewed by two pathologists, and disagreements were resolved by discussion to consensus. CIN2+ was defined as a histological diagnosis of CIN2, CIN3, adenocarcinoma *in situ* or invasive cancer, taken from the structured pathology record. As in routine practice, the pathology request form recorded the referring clinician’s provisional diagnosis, and the histological diagnosis was made on morphological criteria.

### 2.3 Predictors

Fifteen candidate variables recorded at the index visit, before histopathology, were grouped *a priori* into five clinical sources (**Table 1**): demographic and reproductive history (age, menopausal status, gravidity, parity, at least one living child); HPV testing (any high-risk type, HPV16, HPV18, other high-risk types); cytology grade (Bethesda System) (19); colposcopic findings (colposcopic impression, transformation zone type, iodine test negativity and atypical vessels, recorded with the terminology of the International Federation for Cervical Pathology and Colposcopy) (20); and the clinician image-based assessment. The last is a binary variable in which the clinician recorded, at the index visit and before any histopathology result was available, a positive or negative judgement based on the images or the colposcopic examination. It is not derived from the outcome-defining pathology fields and is not a deterministic function of the endpoint; it is nevertheless a near-decision variable whose recording conventions may differ between institutions.

**Table 1:** Candidate variables, coding, *a priori* grouping and univariable discrimination.

Group	Variable	Coding	Univariable AUROC (95% CI)
G1 Demographic and reproductive	Age	Years, continuous	0.519 (0.476–0.563)
	Menopausal status	0 = premenopausal, 1 = postmenopausal	0.502 (0.460–0.544)
	Gravidity	Number of pregnancies, including miscarriage and ectopic pregnancy	0.525 (0.482–0.568)
	Parity	Number of deliveries at ≥28 weeks, including stillbirth	0.503 (0.459–0.546)
	At least one living child	0 = no, 1 = yes	0.493 (0.452–0.535)
G2 HPV	High-risk HPV (any type)	0 = negative, 1 = positive	0.559 (0.518–0.600)
	HPV16	0 = negative, 1 = positive	0.568 (0.524–0.612)
	HPV18	0 = negative, 1 = positive	0.461 (0.419–0.503)
	Other high-risk HPV types	0 = negative, 1 = positive	0.497 (0.457–0.537)
G3 Cytology	Cytology grade (Bethesda)	NILM, ASC-US, ASC-H, LSIL, HSIL, AGC (categorical)	0.628 (0.584–0.671)
G4 Colposcopic findings	Colposcopic impression	Normal, mild, moderate, severe, highly suspicious for CIN3+ (categorical)	0.542 (0.499–0.584)
	Transformation zone type	Type 1 (fully visible), type 2 (partially visible) or type 3 (not visible) (categorical)	0.556 (0.512–0.600)
	Iodine test negativity	0 = no, 1 = yes	0.520 (0.478–0.562)
	Atypical vessels	0 = absent, 1 = present	0.491 (0.449–0.533)
G5 Clinician assessment	Clinician image-based assessment	0 = negative, 1 = positive; judgement recorded by the clinician from the images or the examination	0.671 (0.628–0.715)

All variables were recorded at the index visit, before histopathology. Univariable AUROC: for each held-out fold, discrete variables were scored by the training-fold CIN2+ proportion of their level and age by its training-fold percentile; pooled out-of-fold AUROC with DeLong 95% CI. AGC, atypical glandular cells; ASC-H, atypical squamous cells, cannot exclude HSIL; ASC-US, atypical squamous cells of undetermined significance; HSIL/LSIL, high-/low-grade squamous intraepithelial lesion; NILM, negative for intraepithelial lesion or malignancy.

Missing values were few: age (n = 3), gravidity (n = 3), parity (n = 2) and iodine test result (n = 1); all other variables were complete. Other high-risk HPV types and atypical vessels were recorded in one and two women, respectively, so genotype information beyond HPV16 and HPV18 was effectively not captured. One record contained an implausible age (602 years) and four records had parity exceeding gravidity; these records were retained as recorded so that the fixed protocol was not altered after results were seen, and a sensitivity analysis setting the implausible age to missing is reported. With 242 events and 15 candidate variables (34 parameters after encoding), there were 16.1 events per candidate variable and 7.1 per parameter (21).

### 2.4 Model families

Ten model families were compared: logistic regression (L2, C = 1, balanced class weights); random forest (22); XGBoost (23); LightGBM (24); CatBoost (25); a supervised tabular Transformer (feature-token embeddings of dimension 64, two layers, four heads) (26); FT-Transformer with its published default configuration (8); TabPFN, a tabular foundation model pretrained on a synthetic prior and applied by in-context learning with local weights (10); and Tab-MFM, the same tokenised Transformer pretrained by masked feature reconstruction before supervised fine-tuning (9, 27), evaluated in four configurations that differ only in pretraining (yes/no), column-identity embeddings (on/off) and mask ratio (0.30/0.15). Because pretraining changes the training procedure, the pretrained and non-pretrained Tab-MFM were counted as separate families, giving ten families and twelve configurations; the two further Tab-MFM configurations served only as matched ablations. Hyperparameters were fixed *a priori* from the original implementations or library defaults and were not tuned. The Transformer variants had about 71,000 to 76,000 trainable parameters and FT-Transformer 0.90 million. Early stopping selected epochs 1 to 50; epoch 1 was selected in two FT-Transformer folds, so the neural models may have been under-trained under default settings. Full settings are specified in the configuration files of the code repository.

### 2.5 Validation protocol

A single stratified five-fold split (shuffle, seed 42) was defined once and shared by every model and every analysis, and a single random seed, 13, was used throughout. Within each fold, imputation, encoding, scaling, model fitting and early stopping used the training folds only. Numeric variables were median-imputed and standardised; categorical variables were imputed with the most frequent category and one-hot encoded for the classical learners, and integer-encoded with a vocabulary learned on the training folds for the neural models and TabPFN; early stopping for neural models used an inner stratified 15% split of the training folds. Predictions for each held-out fold were pooled to give one out-of-fold prediction per woman. Neither seeds nor configurations were selected on the basis of results. For external evaluation, logistic regression and the pretrained Tab-MFM were refitted on the full development cohort (Tab-MFM for the median number of epochs selected across folds) and applied without modification.

### 2.6 Performance measures

Discrimination (area under the receiver operating characteristic curve, AUROC, with DeLong 95% confidence intervals (CIs) (28), and area under the precision–recall curve, AUPRC) was computed on uncalibrated pooled out-of-fold predictions, because *post-hoc* calibration is a monotone transformation within each fold and isotonic regression introduces ties that would otherwise mix the choice of calibration method into discrimination. Probability quality (Brier score, expected calibration error over ten equal-width bins, and calibration plots) was assessed on calibrated predictions (29). Calibration used Platt scaling (30) or isotonic regression (31), with the choice nested: for each held-out fold, the method was selected by inner five-fold cross-validation on the remaining folds and fitted on those folds only, so that the held-out labels were never used. For comparison we also computed a pooled procedure, in which one method was chosen per model by the pooled out-of-fold Brier score.

### 2.7 Comparative analyses

Every configuration was compared with logistic regression, with Holm adjustment across the eleven comparisons (32). Three matched ablations isolated pretraining, column-identity embeddings and mask ratio within Tab-MFM, holding architecture, optimisation, seed, folds and preprocessing fixed. Every model family was refitted with and without the clinician image-based assessment. AUROC differences were tested with the DeLong method, and paired differences in AUROC and Brier score were estimated by patient-level bootstrap (2,000 resamples, seed 13) with percentile 95% confidence intervals (33). To test whether the comparisons depended on the fold assignment or the random seed, they were repeated over ten further stratified five-fold cross-validations with new fold assignments and seeds and tested with the corrected repeated cross-validation t test (34), with Holm adjustment; this analysis was specified in writing before it was run. Baseline characteristics were compared between women with and without CIN2+ with Welch’s t test, the Mann–Whitney U test, and χ² or Fisher’s exact tests. As an exploratory analysis, we tested whether the difference between TabPFN and a reference learner changed when the clinician assessment was removed (bootstrap difference-in-differences).

### 2.8 Variable-group attribution

For each model family, all 31 non-empty combinations of the five variable groups were refitted under the same protocol. With v(S) defined as the uncalibrated out-of-fold AUROC of subset S minus 0.5, and v = 0 for the empty set, the exact Shapley value of each group (35) was computed from the 31 subsets. The five values sum to the family’s AUROC minus 0.5, and each group’s share of that total is reported. This is a refitting-based population importance measure (13), not an explanation of one fitted model. Confidence intervals came from 2,000 patient-level bootstrap resamples of the fixed out-of-fold predictions, recomputing all 31 AUROCs in each resample. Agreement of the group rankings across the ten families was summarised by Kendall’s W (36) with a permutation test (10,000 permutations, seed 13), together with whether all families ranked the same group first; these two results were designated in advance as primary, and all others are descriptive. The analysis was specified in writing before any subset model was fitted. For speed, the five neural and foundation-model families were refitted on a graphics processing unit (GPU); the other five families were refitted in the central processing unit (CPU) environment of the main analysis and reproduced it exactly.

### 2.9 Clinician–model complementarity

This analysis was specified in writing before any stratified quantity was computed. It used predictions from models fitted without the clinician assessment, so that the model contributed only routine data. First, discrimination was estimated separately in clinician-positive and clinician-negative women. Second, within each held-out fold, clinician-negative women were ranked by model risk and the top 10%, 20% or 30% were flagged for re-review; this rule uses no outcome labels. We report the proportion of clinician-missed CIN2+ identified, the number expected from flagging the same number of women at random, and pathway sensitivity and specificity. Third, four referral strategies were compared by net benefit (37): the clinician alone (refer if positive); the model alone; a sequential strategy (refer if clinician-positive, otherwise refer if the model’s risk, recalibrated within clinician-negative women using the nested procedure of Section 2.6, reaches the threshold); and an integrated model that includes the clinician assessment as a predictor. The primary learner was logistic regression, with all ten families as a robustness check. Bootstrap confidence intervals were computed for the differences between strategies at threshold probabilities of 0.10, 0.20 and 0.30.

### 2.10 Operating points, decision curves and sample efficiency

Operating points were selected without using the held-out fold: for each fold, the Youden-optimal threshold (38) and the highest threshold achieving at least 95% sensitivity were chosen on inner calibrated predictions of the training folds and applied to the held-out fold; performance was pooled across folds with Wilson confidence intervals. Decision curves covered threshold probabilities of 0.05 to 0.40 (37). Learning curves refitted logistic regression on stratified subsets (20% to 100%) of each training fold, five repetitions per point, using the identical pipeline. Analyses used Python 3.10.11 with scikit-learn 1.7.2, XGBoost 3.1.3, LightGBM 4.7.0, CatBoost 1.2.10, PyTorch 2.5.1 (CPU build), rtdl_revisiting_models 0.0.2 and TabPFN 8.5.0. The analysis code, the fixed fold assignment and random seed, and a synthetic example dataset with the same variable schema are available at https://github.com/SIMON48623/cin2-information-vs-architecture.

## 3 Results

### 3.1 Cohort

Of 879 women, 242 (27.5%) had CIN2 +. Women with and without CIN2+ were similar in age (mean 45.5 and 45.9 years; p = 0.644), menopausal status (31.0% and 35.8% postmenopausal; p = 0.208), gravidity and parity. High-risk HPV (80.6% versus 64.8%) and HPV16 (31.0% versus 14.6%) were more frequent in women with CIN2+ (both p < 0.001), as was ASC-H or HSIL cytology (13.6% versus 2.0%; p < 0.001 for cytology grade). Colposcopic impression was moderate in 61.1% of women and highly suspicious for CIN3+ in 10.9% (p = 0.002 between groups), and transformation zone type also differed (p < 0.001); no woman had a normal colposcopic impression. The full baseline table, overall and by CIN2+ status, is available in the code repository. The clinician assessment was positive in 197 women, of whom 122 (61.9%) had CIN2+; of the 682 clinician-negative women, 120 (17.6%) had CIN2 +. The external cohort included 103 women with 34 (33.0%) CIN2 +.

### 3.2 No model family outperformed logistic regression

Discrimination ranged from 0.726 to 0.761 across the twelve configurations (**Table 2**). Logistic regression reached 0.757 (95% CI 0.720–0.795). No configuration outperformed it (**Figure 2a**; **Table 2**): the differences were +0.004 for TabPFN (95% CI −0.008 to 0.016; p = 0.554) and −0.002 for CatBoost (−0.026 to 0.022; p = 0.860), and every other configuration was numerically lower. The pretrained Tab-MFM reached 0.737 (−0.021 versus logistic regression; p = 0.062). After Holm adjustment no comparison was significant (smallest adjusted p = 0.116). The repeated cross-validations gave the same result: no configuration differed significantly from logistic regression (all Holm-adjusted p = 1.00; **Table 2**), and the largest mean gain, +0.010 AUROC for TabPFN (corrected p = 0.15), was well below the 0.041 to 0.072 lost when the clinician assessment was removed (Section 3.4). Probability quality differed slightly: the Brier score of TabPFN was lower than that of logistic regression (−0.0068, 95% CI −0.0118 to −0.0020; p = 0.008), without a corresponding difference in discrimination.

**Table 2:** Discrimination, probability quality and paired comparison with logistic regression (full predictor set).

Model	AUROC (95% CI)	AUPRC	Brier score	ECE	ΔAUROC vs logistic regression (95% CI)	p (Holm)	Repeated CV ΔAUROC, mean (range)
TabPFN	0.761 (0.723–0.799)	0.610	0.158	0.015	+0.004 (−0.008 to 0.016)	1.000	+0.010 (−0.001 to 0.018)
Logistic regression	0.757 (0.720–0.795)	0.567	0.165	0.034	Reference	—	Reference
CatBoost	0.755 (0.717–0.793)	0.592	0.157	0.013	−0.002 (−0.026 to 0.022)	1.000	+0.000 (−0.019 to 0.008)
Tab-MFM, no column identity	0.749 (0.711–0.786)	0.557	0.166	0.034	−0.008 (−0.026 to 0.010)	1.000	−0.005 (−0.028 to 0.012)
Tabular Transformer	0.744 (0.706–0.782)	0.570	0.169	0.030	−0.013 (−0.030 to 0.004)	0.886	−0.006 (−0.024 to 0.011)
Tab-MFM, no pretraining	0.743 (0.704–0.781)	0.587	0.166	0.050	−0.015 (−0.035 to 0.005)	0.911	−0.014 (−0.048 to 0.011)
XGBoost	0.740 (0.701–0.778)	0.569	0.165	0.031	−0.017 (−0.043 to 0.007)	0.911	−0.008 (−0.020 to 0.002)
FT-Transformer	0.739 (0.701–0.778)	0.551	0.171	0.040	−0.018 (−0.043 to 0.007)	0.911	−0.009 (−0.046 to 0.008)
Tab-MFM, pretrained (mask 0.30)	0.737 (0.698–0.775)	0.555	0.171	0.043	−0.021 (−0.042 to 0.001)	0.617	−0.010 (−0.045 to 0.004)
Random forest	0.731 (0.693–0.770)	0.554	0.168	0.034	−0.026 (−0.054 to 0.005)	0.650	−0.017 (−0.038 to −0.004)
LightGBM	0.729 (0.690–0.767)	0.545	0.170	0.022	−0.029 (−0.060 to 0.002)	0.617	−0.027 (−0.053 to −0.006)
Tab-MFM, pretrained (mask 0.15)	0.726 (0.687–0.765)	0.554	0.171	0.026	−0.031 (−0.056 to −0.008)	0.116	−0.016 (−0.053 to 0.016)

Pooled out-of-fold predictions, n = 879. AUROC and AUPRC from uncalibrated predictions; Brier score and expected calibration error (ECE, ten equal-width bins) from nested-calibrated predictions. ΔAUROC = configuration minus logistic regression, with 95% confidence intervals from 2,000 paired patient-level bootstrap resamples; p values from DeLong tests with Holm adjustment across the eleven comparisons. Repeated CV: mean (range) of ΔAUROC across ten repetitions of stratified five-fold cross-validation with new fold assignments and seeds; corrected repeated cross-validation t tests, all Holm-adjusted p = 1.00.

**Figure 2:**
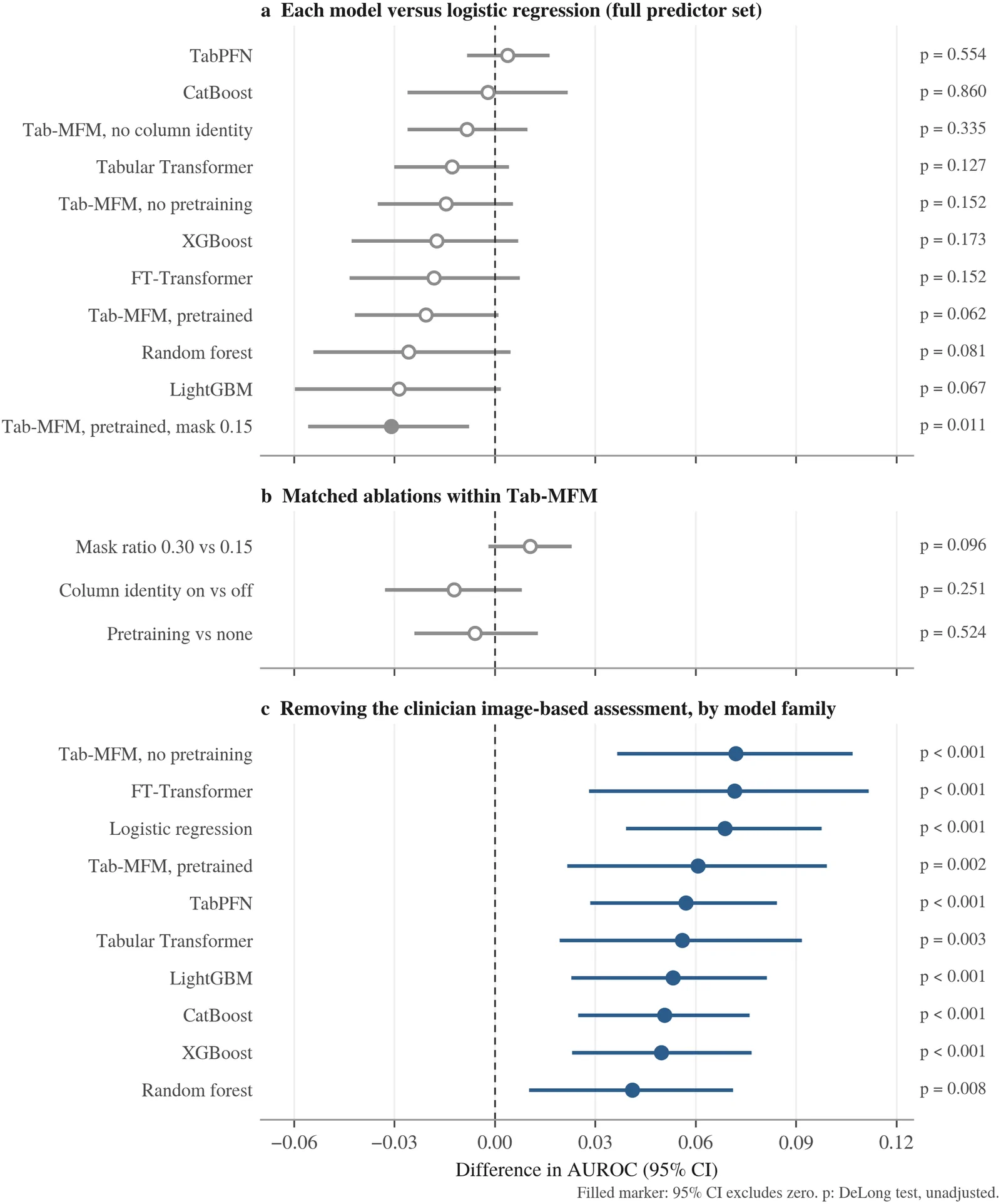
Effect sizes on a common scale. Differences in out-of-fold AUROC with paired 95% bootstrap confidence intervals. **(a)** Each configuration versus logistic regression. **(b)** Matched ablations within Tab-MFM. **(c)** Effect of removing the clinician image-based assessment in each model family. Filled markers indicate intervals that exclude zero; p values are from unadjusted DeLong tests.

### 3.3 Matched ablations were null

None of the three matched ablations within Tab-MFM was detectable (**Figure 2b**): pretraining −0.006 (95% CI −0.024 to 0.013; p = 0.524), column-identity embeddings −0.012 (−0.033 to 0.008; p = 0.251) and mask ratio 0.30 versus 0.15 + 0.010 (−0.002 to 0.023; p = 0.096); Brier-score differences were likewise not detectable (all p ≥ 0.183).

### 3.4 The clinician assessment mattered in every model family

Removing the clinician image-based assessment reduced AUROC in all ten model families, by 0.041 to 0.072 (all p ≤ 0.008; **Figure 2c**); for logistic regression the reduction was 0.069 (95% CI 0.039 to 0.098). Brier scores worsened in every family (all p ≤ 0.037). Without it, AUROC ranged from 0.667 to 0.704. The smallest of these reductions exceeded the largest architectural effect in absolute size. In univariable nested analysis (**Table 1**), the clinician assessment was the most discriminating single variable (AUROC 0.671, 95% CI 0.628–0.715), followed by cytology grade (0.628) and HPV16 (0.568).

In the exploratory analysis, TabPFN did not differ from logistic regression with the clinician assessment (+0.004; p = 0.554) or without it (+0.015, 95% CI −0.001 to 0.032; p = 0.065); the change between the two settings was +0.012 (−0.002 to 0.025; p = 0.081), and relative to CatBoost it was −0.006 (−0.024 to 0.013; p = 0.496).

### 3.5 Every model family attributed discrimination in the same order

All ten families ranked the five groups identically (**Figure 3**): the clinician assessment first, followed by cytology, HPV testing, colposcopic findings, and demographic and reproductive history. Kendall’s W was 1.00 (permutation p = 0.0001, the smallest value attainable with 10,000 permutations; the largest W among permutations was 0.49). The clinician assessment accounted for 39% to 50% of each family’s discrimination above chance (Shapley value 0.095 to 0.119), cytology for 20% to 25%, HPV testing for 17% to 20% and colposcopic findings for 10% to 15%; for these four groups the confidence interval excluded zero in every family. Demographic and reproductive history contributed −3% to 9%, with intervals including zero in 9 of ten families.

**Figure 3:**
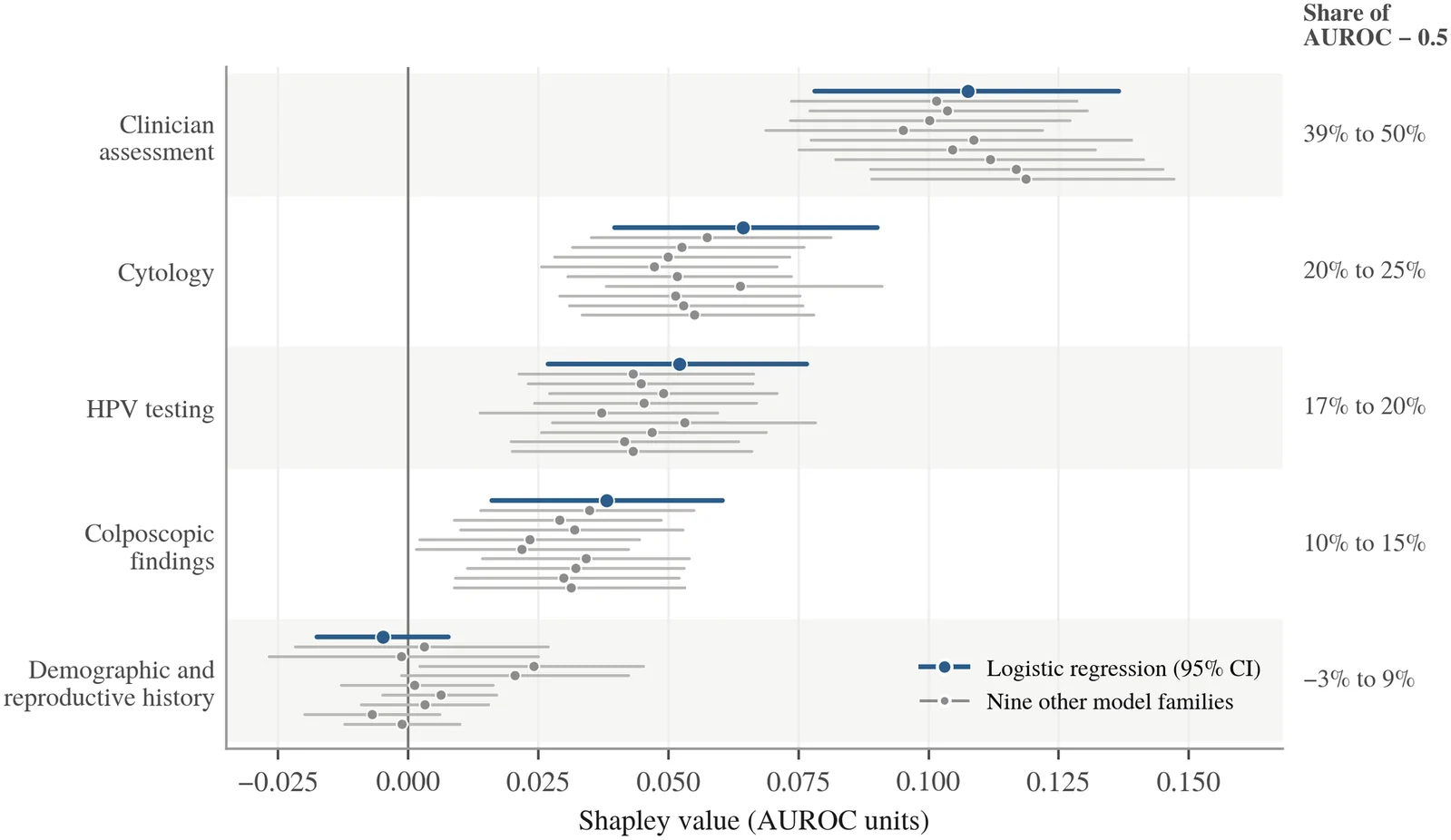
Exact Shapley attribution of discrimination to five variable groups in ten model families. Within each group, each row is one model family (logistic regression in blue with its 95% bootstrap interval; nine other families in grey). Values are in AUROC units and sum to AUROC − 0.5 within each family; the right-hand column gives the range of each group’s share across families. Groups are ordered by rank, which was identical in all ten families.

For logistic regression, the Shapley value of the clinician assessment (0.108, 95% CI 0.078 to 0.136) was larger than the reduction in AUROC when it was removed from the full set (0.069; Section 3.4). Part of its information is therefore shared with the cytology, HPV and colposcopic groups. In descriptive checks, all ten families ranked the clinician assessment first in 95.9% of bootstrap resamples, and its Shapley value exceeded that of cytology with an interval excluding zero in 9 of ten families (TabPFN: +0.041, 95% CI −0.003 to 0.080).

Refitting on different hardware changed the neural models by amounts comparable to the architectural contrasts. Relative to the main analysis, the full-set AUROC of the five refitted neural and foundation-model families differed by −0.019 to +0.004 (FT-Transformer 0.720 versus 0.739), and individual predicted probabilities differed by up to 0.31; the five classical families reproduced the main analysis exactly. The AUROC of every subset in every family is available in the code repository, so that the attribution can be recomputed.

### 3.6 Routine data complemented the clinician in clinician-negative women

The clinician assessment alone had sensitivity 0.504, specificity 0.882, positive predictive value 0.619 and negative predictive value 0.824; 120 of the 242 CIN2+ cases (49.6%) occurred in clinician-negative women. Without the clinician assessment, models discriminated modestly within each stratum: among clinician-negative women AUROC ranged from 0.622 to 0.645 across the ten families (logistic regression 0.643, 95% CI 0.589–0.696), and among clinician-positive women from 0.597 to 0.676, with no difference between strata in any family (all p ≥ 0.46).

Re-reviewing clinician-negative women in order of logistic-regression risk identified clinician-missed CIN2+ at 1.6 to 1.8 times the random rate (**Table 3**; **Figure 4a**). Flagging the top 10% (70 women) identified 22 of 120 (18.3%, 95% CI 11.8–25.6%; 12 expected at random); the top 20% (136 women) identified 41 (34.2%, 25.7–42.7%; 24 expected); and the top 30% (205 women) identified 56 (46.7%, 37.9–55.5%; 36 expected). Pathway sensitivity rose from 0.504 to 0.595, 0.674 and 0.736, while specificity fell from 0.882 to 0.807, 0.733 and 0.648. Across the ten families, the yield at 20% ranged from 29.2% to 35.0%.

**Table 3:** Re-review of clinician-negative women ranked by logistic-regression risk.

Strategy	Women re-reviewed	Missed CIN2+ found, n (%; 95% CI)	Expected at random	Re-reviews per case	Pathway sensitivity (95% CI)	Pathway specificity (95% CI)
Clinician alone	—	—	—	—	0.504	0.882
Clinician, then re-review top 10% of clinician-negative women	70	22 (18.3%; 11.8–25.6%)	12	3.2	0.595 (0.534–0.656)	0.807 (0.776–0.837)
Clinician, then re-review top 20% of clinician-negative women	136	41 (34.2%; 25.7–42.7%)	24	3.3	0.674 (0.615–0.732)	0.733 (0.699–0.768)
Clinician, then re-review top 30% of clinician-negative women	205	56 (46.7%; 37.9–55.5%)	36	3.7	0.736 (0.680–0.788)	0.648 (0.611–0.685)

Clinician-negative women: n = 682, of whom 120 had CIN2 +. The model was fitted without the clinician assessment; ranking was done within each held-out fold and uses no outcome labels. Results for the other nine model families are shown in **Figure 4a**.

**Figure 4:**
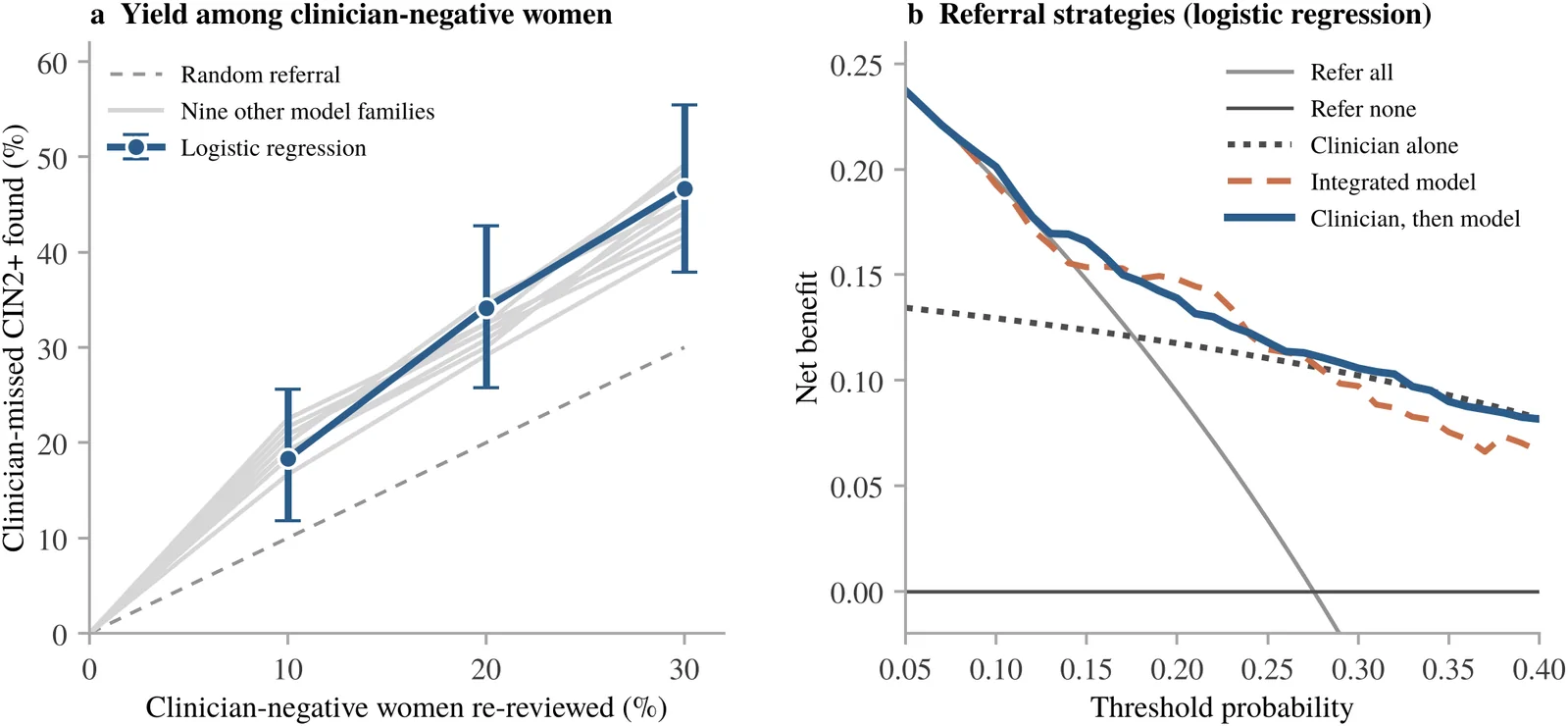
Clinician–model complementarity. **(a)** Proportion of clinician-missed CIN2+ identified by re-reviewing the top 10%, 20% or 30% of clinician-negative women ranked by model risk (logistic regression with 95% bootstrap intervals; grey lines, nine other model families; dashed line, random referral). **(b)** Net benefit of three of the four referral strategies (Section 2.9), based on logistic regression: the clinician alone, the integrated model and the sequential strategy (clinician, then model); the model-alone strategy is not shown, and its values are in the code repository.

The sequential strategy gave higher net benefit than the clinician alone at threshold probabilities of 0.10 (+0.072, 95% CI 0.048 to 0.096) and 0.20 (+0.021, 0.004 to 0.041) but not at 0.30 (+0.003, −0.007 to 0.015) (**Figure 4b**). The advantage at 0.10 held in 10 of 10 families, at 0.20 in 5, and at 0.30 in 0. At 0.10, the sequential strategy was only marginally better than referring every woman (+0.006), so its gain over the clinician alone at low thresholds reflects the clinician’s low sensitivity rather than a large benefit of the model. The sequential strategy and the integrated model performed similarly (0.10: +0.009, 0.001 to 0.017; 0.20: −0.009, −0.021 to 0.002; 0.30: +0.008, −0.003 to 0.019).

In the external cohort (descriptive only), the clinician assessment had sensitivity 0.500 and specificity 0.826, close to the development cohort. Among 74 clinician-negative women with 17 CIN2+, flagging the top 10%, 20% and 30% by the frozen logistic model identified 2, 4 and 7 cases, against 1.7, 3.4 and 5.1 expected at random.

### 3.7 Calibration, operating points and decision curves

After nested calibration, Brier scores were 0.165 for logistic regression, 0.157 for CatBoost and 0.158 for TabPFN (**Figure 5a**). Nesting the choice of calibration method changed Brier scores little relative to pooled selection, which understated them by a median of 0.0013 (maximum 0.0049) across 22 configurations; for the pretrained Tab-MFM the difference was +0.0021 (95% CI 0.0000 to 0.0041). Within clinician strata, the ratio of observed to expected CIN2+ cases was 0.93 in clinician-negative and 1.06 in clinician-positive women for logistic regression, and 1.00 and 1.00 for TabPFN.

**Figure 5:**
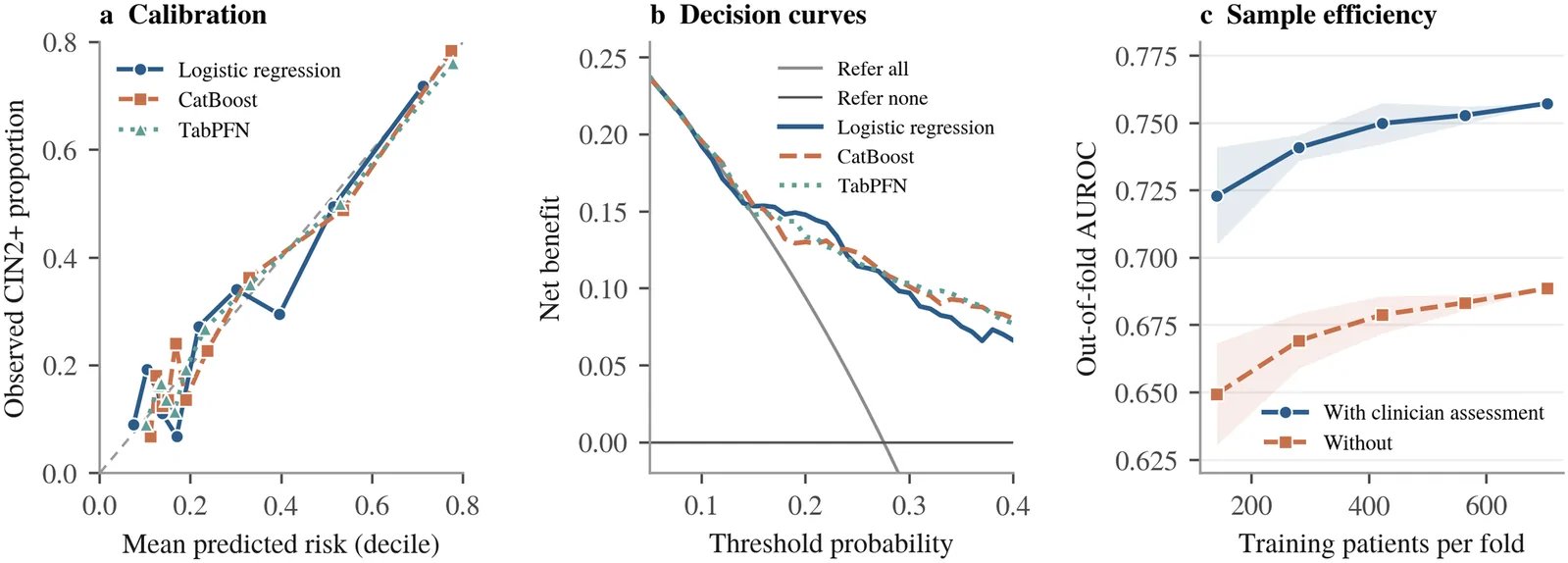
Calibration, clinical utility and sample efficiency. **(a)** Observed CIN2+ proportion against mean predicted risk by decile of nested-calibrated predictions for three reference models. **(b)** Decision curves for the same models across threshold probabilities of 0.05 to 0.40. **(c)** Learning curves for logistic regression with and without the clinician image-based assessment: mean out-of-fold AUROC across five repetitions, with shading showing ±1 standard deviation.

With thresholds chosen without the held-out fold, the Youden rule for logistic regression (median threshold 0.221) gave sensitivity 0.711 (95% CI 0.651–0.764) and specificity 0.684 (0.647–0.719), flagging 42.4% of women. The high-sensitivity rule (median threshold 0.099) gave sensitivity 0.967 (0.936–0.983) at specificity 0.146 (0.121–0.176), flagging 88.5%. Both are exploratory operating points, not validated decision thresholds.

All three reference models exceeded referring every woman above a threshold probability of about 0.15 (**Figure 5b**). Differences between them were small and not consistent in direction: at 0.20 logistic regression had higher net benefit than CatBoost (−0.018, 95% CI −0.034 to −0.002) and TabPFN (−0.015, −0.026 to −0.003), with no difference at 0.10 or 0.30.

### 3.8 Sample efficiency and sensitivity analysis

Logistic regression gained +0.016 in AUROC as training size rose from 281 to 703 women per fold, and +0.019 without the clinician assessment (**Figure 5c**). Returns diminished with size, and the gap between the two curves stayed between 0.069 and 0.074 at every size. Setting the implausible age to missing changed the AUROC of logistic regression from 0.757 to 0.759 (from 0.689 to 0.691 without the clinician assessment).

### 3.9 External cohort

In the external cohort, frozen logistic regression reached AUROC 0.710 (95% CI 0.600–0.820) and frozen Tab-MFM 0.716 (0.606–0.826); without the clinician assessment the values were 0.682 and 0.693. With 34 events, none of these differences was detectable (clinician assessment, logistic regression: +0.028, p = 0.567), and the external results are descriptive.

## 4 Discussion

Under one fixed protocol, ten model families, from logistic regression to a pretrained foundation model, discriminated CIN2+ almost equally well, and none improved on logistic regression. What mattered was the information available. Removing the clinician’s image-based assessment lowered discrimination in every family, and all ten families attributed their discrimination to the same sources in the same order: the clinician assessment, cytology, HPV testing, colposcopic findings and, last, demographic and reproductive history. In this retrospective cohort, among women whom the clinician judged negative, routine data identified missed CIN2+ at 1.6 to 1.8 times the rate expected from random re-review.

These results agree with a systematic review that found no general performance benefit of machine learning over logistic regression for clinical prediction (6). They are also consistent with benchmarks in which deep tabular models seldom surpass well-configured baselines (7). A tabular foundation model (10) matched logistic regression in discrimination and had a slightly lower Brier score, a modest gain in probability quality rather than in ranking. This conclusion was robust to the choice of fold assignment and random seed: across ten repeated cross-validations no configuration improved significantly on logistic regression, and the largest gain, about 0.01 AUROC for the foundation model, was a quarter of the smallest loss caused by removing the clinician assessment. Like any comparison made in a single cohort, it describes the evaluated protocol rather than every possible setting. Matched protocols matter as well. Refitting the same neural networks on different hardware changed individual predictions by up to 0.31, and on tables of this size, variation between runs is of the same order as the gains typically claimed for new architectures (11). For deep learning in cervical cancer care, the practical implication is that a new architecture should be compared with logistic regression under a shared protocol and with paired uncertainty before an improvement is claimed.

The ordering of information is clinically coherent. The clinician’s assessment integrates the colposcopic examination with the referral findings, so it is not surprising that it carried the largest share. Any information that the assessment shares with the reference standard, as when pathologists read the provisional diagnosis on the request form, applies to every model family alike and so cannot favour any architecture. Cytology and HPV testing, the results on which current risk-based management is built (2), ranked next in every family, whereas structured colposcopic findings contributed less. This modest share should not be read as a verdict on colposcopy: the clinician assessment draws on the same examination, Shapley values divide the information that overlapping groups share, and in this cohort the most severe colposcopic category had a lower CIN2+ proportion (20/96) than the moderate category (173/537). Because the ranking was identical across learners with very different inductive biases, it behaves as a property of the data rather than of a particular model, which explanations of a single fitted model, such as integrated gradients or SHAP, cannot establish (12). The ranking depends on the *a priori* grouping and on AUROC as the measure of value, and agreement between learners fitted to the same data shows that it does not depend on the learner, not that it transfers unchanged to other populations.

Half of the CIN2+ cases in this cohort occurred in women judged negative by the clinician, and this is the gap that routine data might help to close. In retrospective data, a sequential rule, in which the clinician decides first and a model built from routine data selects clinician-negative women for re-review, found about a third of the missed cases when a fifth of these women were re-reviewed. It performed as well as an integrated model and keeps the clinician’s judgement visible. Its net-benefit gain over the clinician alone was concentrated at low threshold probabilities, so its value lies in shifting the balance between sensitivity and workload, and whether that shift is worthwhile depends on local resources and on how flagged women are managed. Larger gains are more likely to come from richer information than from more complex learners. The strongest published CIN2+ models use full HPV genotyping in large screening cohorts (5), whereas genotype information beyond HPV16 and HPV18 was not captured in our records and colposcopic images are not represented in structured data at all; the learning curves likewise showed diminishing returns from additional patients.

The study has several strengths. All ten model families were compared under one fixed protocol with paired uncertainty, both new analyses were specified in writing before they were computed, and the analysis code and a synthetic dataset are publicly available. It also has limitations. The development cohort is retrospective and from a single centre, and the external cohort, with 34 events, supports only descriptive conclusions and is too small for a stable assessment of generalisability or calibration, so any use in another population would require recalibration. Hyperparameters were fixed rather than tuned, which keeps comparisons matched but may understate the attainable performance of every model, including logistic regression. Finally, the findings concern one endpoint and one set of routinely recorded variables, and should be confirmed in other populations.

## 5 Conclusion

In women referred for colposcopy, routinely recorded structured variables supported moderate discrimination for CIN2+, and neither gradient boosting, deep tabular networks, self-supervised pretraining nor a pretrained foundation model improved significantly on logistic regression. The largest and most consistent contribution came from the clinician’s own assessment, followed by cytology and HPV testing, in the same order for every learner; in retrospective data, routine data added to the clinician modestly in clinician-negative women. Progress in this setting is more likely to come from richer information than from more complex models, and claims for new architectures should be tested against logistic regression with paired uncertainty across repeated data splits. Prospective evaluation would be required before any clinical use.

## Data Availability

The original contributions presented in the study are included in the article/supplementary material. Further inquiries can be directed to the corresponding author.
